# Novel Quantitative Real-Time LCR for the Sensitive Detection of SNP Frequencies in Pooled DNA: Method Development, Evaluation and Application

**DOI:** 10.1371/journal.pone.0014560

**Published:** 2011-01-19

**Authors:** Androniki Psifidi, Chrysostomos Dovas, Georgios Banos

**Affiliations:** 1 Department of Animal Production, Faculty of Veterinary Medicine, Aristotle University of Thessaloniki, Thessaloniki, Greece; 2 Laboratory of Microbiology and Infectious Diseases, Faculty of Veterinary Medicine, Aristotle University of Thessaloniki, Thessaloniki, Greece; Texas A & M University, United States of America

## Abstract

**Background:**

Single nucleotide polymorphisms (SNP) have proven to be powerful genetic markers for genetic applications in medicine, life science and agriculture. A variety of methods exist for SNP detection but few can quantify SNP frequencies when the mutated DNA molecules correspond to a small fraction of the wild-type DNA. Furthermore, there is no generally accepted gold standard for SNP quantification, and, in general, currently applied methods give inconsistent results in selected cohorts. In the present study we sought to develop a novel method for accurate detection and quantification of SNP in DNA pooled samples.

**Methods:**

The development and evaluation of a novel Ligase Chain Reaction (LCR) protocol that uses a DNA-specific fluorescent dye to allow quantitative real-time analysis is described. Different reaction components and thermocycling parameters affecting the efficiency and specificity of LCR were examined. Several protocols, including gap-LCR modifications, were evaluated using plasmid standard and genomic DNA pools. A protocol of choice was identified and applied for the quantification of a polymorphism at codon 136 of the ovine *PRNP* gene that is associated with susceptibility to a transmissible spongiform encephalopathy in sheep.

**Conclusions:**

The real-time LCR protocol developed in the present study showed high sensitivity, accuracy, reproducibility and a wide dynamic range of SNP quantification in different DNA pools. The limits of detection and quantification of SNP frequencies were 0.085% and 0.35%, respectively.

**Significance:**

The proposed real-time LCR protocol is applicable when sensitive detection and accurate quantification of low copy number mutations in DNA pools is needed. Examples include oncogenes and tumour suppressor genes, infectious diseases, pathogenic bacteria, fungal species, viral mutants, drug resistance resulting from point mutations, and genetically modified organisms in food.

## Introduction

Single Nucleotide Polymorphisms (SNP) are the most common type of genetic markers and refer to a position where two alternative bases occur at appreciable frequency (>1%) in a population [1]. The identification of SNP is fundamental to basic life science, human hereditary disease diagnosis and pharmacogenomics [2], [3]. SNP detection is also of great importance to agriculture and animal science, addressing quantitative trait loci (QTL) identification, marker assisted selection, genome mapping, food traceability, parental tests, detection of genetically modified organisms in food and feed, diagnosis of animal genetic diseases, and disease susceptibility.

Typically, in genetic diseases caused by mutations in high proportion of DNA, sensitivity of diagnostic assays is not a critical factor for SNP detection. However, in cases where mutated DNA molecules correspond to a small fraction of the wild-type DNA, for example oncogenes or tumour suppressor genes and microbial or viral mutations associated with drug resistance, the sensitivity of SNP detection becomes a critical factor [4].

Estimation of allele frequency is required in a number of applications of great interest that use pooled DNA samples, such as monitoring minimal residual disease in the course of malignant haematopathies and the quantitative assessment of post transplant chimerism. Association studies of non-Mendelian complex diseases have emphasized cost benefits of allele frequency estimation in DNA pools [5] and raised the need for reliable, highly sensitive, fast throughput screening assays. In addition, DNA pooling can reduce the genotyping effort and cost. For example, in milking ruminants, bulk milk (meaning the total amount of milk produced and collected on a given day in a certain farm) has already been used as a source of DNA pooled across all animals raised on the farm, for selective genotyping in marker assisted selection studies, QTL detection and gene mapping projects [6], [7].

Current methods of SNP detection differ in accuracy, sensitivity and throughput. Traditional procedures such as restriction fragment length polymorphism (RFLP), denaturation gradient gel electrophoresis and chemical cleavage of mismatched heteroduplexes are labour intensive, non-automated processes and, most importantly, lack sensitivity [4]. Therefore, a large number of more sensitive and selective approaches for SNP detection have been introduced in recent years. In general, each method bases SNP detection on one of the following procedures: hybridization, invasive cleavage, oligonucleotide ligation, and primer extension using either allele-specific nucleotide incorporation or allele-specific Polymerase Chain Reaction (PCR) [8]. The products of such procedures are usually analyzed with gel separation, arrays, mass spectrometry, or fluorescence plate reader techniques [9]. Some of these techniques have been also found suitable for the quantification of SNP. These include fluorescence in situ hybridization, denaturing HPLC [10], bioluminometric assay [11], real-time PCR [3], oligo-ligation assay, Invader assay™ (Third Wave Technologies Inc), DNA chips [12] and Pyrosequencing [13]. To date, however, there is not a generally accepted golden standard method for SNP quantification, and, in general, currently applied methods give inconsistent results in selected cohorts.

Real-time PCR has been extensively used for the detection and quantification of SNP in DNA pools. Detection and quantification limits of this technique vary among individual SNP, ranging between 1% to 25% [14], [15]. Nevertheless, such limits are not adequate in cases of testing blood for early detection of cancer, monitoring disease progression and assessing response to therapy [16], [17], where sensitivity at levels greater than 1∶1000 might be required.

Ligase Chain Reaction (LCR) is another DNA amplification method developed as an alternative to PCR [18]. LCR evolved as a very promising diagnostic technique that is often utilized in conjunction with a primary PCR amplification. LCR employs a thermostable ligase and allows the discrimination of SNP. Two complementary pairs of DNA oligonucleotides are utilized and exponential signal amplification analogous to PCR is achieved. However, although conventional LCR is characterized by highly specificity of ligation, background ligation in the absence of template may still occur at a low level [19]. In order to prevent this template-independent ligation, another LCR protocol, gap-LCR, has been developed [18], [20]. Gap-LCR utilizes a DNA polymerase to seal a gap at the site of point mutation between adjacent oligonucleotides and a DNA ligase, to seal the nick between them [18], [4], [20]. However, the fact that, to this date, both LCR and gap-LCR are gel based separation methods limits their use for high throughput applications [21].

In recent years, new methods that combined LCR with detection systems thereby alleviating the laborious gel separation problem have been reported. For example, labelling of LCR oligonucleotides with biotin and digoxigenin, combined with an ELISA-based detection system permitted sensitivity increase and ease of LCR applications [22]. Increased sensitivity was also attained when asymmetric gap-LCR products were detected by microparticle enzyme immunoassay [23]. A gap-LCR assay has also been combined with a fluorescence polystyrene microsphere measurement platform that allows multiplex analysis of SNP [24].

Despite several reports of improvements in LCR detection systems, these refer mainly in the sensitivity of SNP detection and not the quantification potential of LCR. Only recently, a semi-quantitative real-time gap-LCR assay, coupled with PCR, for the sensitive detection of p53 mutation at low levels in surgical margins of tumours was reported [19]. In this case, oligonucleotides labelled with FAM and TAMRA were used for visualization of the LCR products during amplification [19]. This assay required modification of the real-time platform to accommodate excitation of FAM at 494 nm and detection of TAMRA emission at 605 nm. In addition, purification and quantification of the PCR amplicons was required before they could be used as templates for the LCR.

The objective of this study was to develop a quantitative real-time LCR-based method for the sensitive detection and accurate quantification of SNP in DNA pools suitable for routinely performed high throughput diagnostics. This novel protocol combined the advantages of LCR detection with the use of a DNA binding fluorescent dye to allow quantitative real-time analysis. The effect of different parameters, such as reaction components, thermocycling conditions and oligonucleotide thermodynamic properties, in the specificity and efficiency of this real-time LCR protocol were studied. Modifications of the method (gap-LCR) were also introduced and evaluated. An ovine SNP associated with susceptibility to scrapie was used as a model in this study. Scrapie is a fatal degenerative disorder of small ruminants' central nervous system that belongs to the transmissible spongiform encephalopathies. More specifically, a mutation at codon 136 of the ovine *PRNP* gene, which changes from GCC to GTC, coding alanine (A_136_) and valine (V_136_), respectively, and increases susceptibility to the disease, was detected and quantified in plasmid and genomic DNA pools. The allele V_136_ was selected because it is usually detected at very low frequencies depending mainly on the breed of sheep. In the present study, milk samples from the highest milk producing and most prolific Greek dairy sheep breed, Chios, were examined, where a 0.4% V_136_ frequency across the population has been reported [25].

## Materials and Methods

### Sample collection and individual genotyping

Individual milk samples were taken from 60 ewes of the Chios dairy breed of sheep raised in 5 different flocks at the northern part of Greece. Milk samples were collected in 50 ml tubes in the milking parlour, under aseptic conditions, and were immediately placed in isothermic boxes and transferred to the laboratory. Milk sampling took place during the routine milking process in the farm and no animals were treated, handled or otherwise inconvenienced.

Genomic DNA was isolated from these milk samples using a commercial kit, Nucleospin® Blood (Mackerey-Nagel, Duren, Germany), modified properly for milk conditions [26]. All samples were genotyped for the *PRNP* gene using a RFLP analysis protocol [27], modified by using a different upstream primer PrPov1: GTCAAGGTGGTAGCCACA. The specificity of RFLP analysis was verified by sequencing 21 of these samples.

### Preparation of plasmid and genomic standard pools

Primers CLPRNP-F (5′-CAT GAA GCA TGT GGC AGG AGC TG-3′) and CLPRNP-R (5′- ACC ACT ACA GGG CTG CAG GTA GAC-3′) were used to amplify a 261 base pair (bp) fragment of the *PRNP* gene of a previously detected heterozygous ewe, containing the mutant V_136_ and the wild-type A_136_ allele. The amplicons were cloned into PCR®II-TOPO® vector (Invitrogen, Karlsruhe, Germany). Plasmid DNA was extracted using the Nucleospin Plasmid kit (Macherey-Nagel, Düren, Germany) and linearized with NotI-HF™ (New England Biolabs Inc., MA, USA). Selection of clones containing V_136_ or A_136_ was based on restriction analysis using BspHI (New England Biolabs Inc., MA, USA). The concentration of plasmid DNA samples was determined by spectrophotometry. Stocks from two selected plasmid clones (V_136_ and A_136_) were diluted with 10 mM Tris buffer to obtain solutions containing 10^11^ copies/ml. DNA pools with a total number of 5·10^9^ copies/ml, defined as mutant DNA frequencies of 100%, 25%, 6.25%, 1.56% and 0.39%, were generated by serial dilutions of mutant V_136_ plasmid DNA in 1∶4 ratios with the wild-type A_136_ plasmid DNA. These pooled plasmid DNA samples were used to obtain a standard curve, in order to assess linearity and amplification efficiency, variability, dynamic range of quantification, and detection limit of the method described below.

In addition, genomic DNA pools were prepared from extracted samples containing the V_136_ polymorphism at different pre-determined frequencies (50%, 25%, 6.25%, 1.56%, and 0.39%). These were created by mixing equimolar genomic DNA extracts from homozygous V_136_ (mutant DNA) and A_136_ (wild-type) individuals that were quantified by spectrophotometry. For each frequency, four different DNA pools were prepared using extracts from different individuals. These genomic DNA pools were used to test the accuracy of quantification of the method described below.

### Method description for SNP detection and quantification

#### Basic protocol

**Figure 1:**
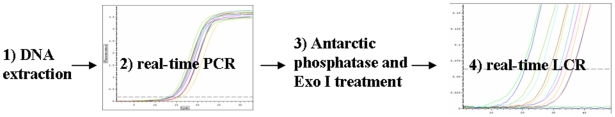
Schematic representation of the entire process for SNP detection and quantification.

A quantitative real-time LCR protocol was developed to assess the prevalence of mutant (V_136_) polymorphism in extracts from DNA pools (genomic or plasmid) and natural extracts from individual ovine milk samples. The full process is illustrated in Figure 1.

**Figure 2:**
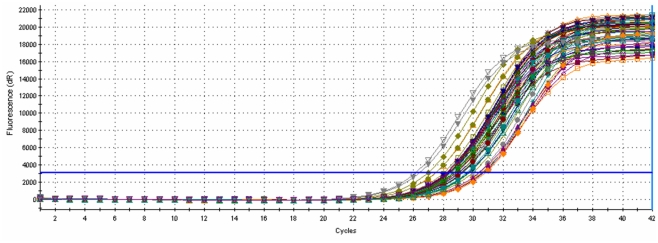
Real-time PCR amplification curves of genomic DNA isolated from different animals.

Prerequisite for accurate quantification of mutant DNA by real-time LCR was the different templates to be equimolar. For this reason, after the isolation of genomic DNA, a real-time PCR which amplified a genomic region containing the SNP was introduced (Figure 2). Due to the PCR amplification plateau effect, it was expected that the final PCR products would be equimolar irrespectively of the initial DNA concentration of the different DNA extracts. The use of a DNA intercalating dye (EvaGreen) permitted the monitoring of concentration of the PCR products [28], [29]. For this purpose, two primers, PRNPf (GGCCTTGGTGGCTACATGCTGGGA) and PRNPr (CCCTTGGTGGTGGTGGTGACTGTGTG) targeting a 216 bp *PRNP* genomic region were designed. One µl of plasmid or extracted DNA was added to a 20 µl reaction mixture. The PCR reactions were optimized for standard cycling conditions with an initial denaturation step at 95°C (3 min), followed by 42 cycles of denaturation at 95°C (30 s) and annealing at 65°C (1 min), using the Mx3005P QPCR system (Stratagene Co., La Jolla, CA). Optimal reaction conditions for PCR were determined as follows: 0.5 units of Platinum® *Taq* DNA polymerase (Invitrogen, The Netherlands), 2 µl PCR buffer (10X), 200 µM each dATP, dCTP, dGTP, and dTTP, 2 mM MgCl_2_, 1 µl DNA-specific fluorescent dye EvaGreen™ (Biotium, Hayward, CA, USA) (20X), 0.2 mM PRNPf and 0.2 mM PRNPr gene specific primers (Table 1), and water up to 20 µl. All samples were run in triplicates. The plateau of the amplification curve for each sample was required to lie within three standard deviations of the mean. Samples with a plateau that deviated from the mean by more than three standard deviations were excluded from the real-time LCR trials and were re-amplified in a subsequent PCR.

**Table 1:** Oligonucleotides used in the different real-time LCR trials.

Oligonucleotide	Sequence 5′→3′	*T_m_* a
LCPR1 b	CACTTCCCAGCATGTAGCCAC	68.6°C
LCPR2	GTGGCTACATGCTCGGAAGTGT	69.6°C
LCPR3S	AAGAGGCCTGCTCATGA	66.7°C
LCPR4S b	CATGAGCAGGCCTCTT	65.0°C
LCPR2G	GTGGCTACATGCTCGGAAGTG	68.9°C
LCPR3G	AAGAGGCCTGCTCATG	65.0°C
LCPR3L	CAAAATGTATAAGAGGCCTGCTCATGA	69.8°C
LCPR4L b	CATGAGCAGGCCTCTTATACATTTTG	68.9°C

The nucleotides which complement to the mutant SNP target are underlined on the discriminating oligonucleotides.

a The melting temperature *T_m_* was estimated using the DINAMelt web server (http://www.bioinfo.rpi.edu/applications/hybrid/hybrid2.php).

b 5′ phosphorylation on the ligating oligonucleotide.

A treatment of PCR products with Exonuclease I and Antarctic Phosphatase followed in order to digest all single stranded DNA (PCR products and primers) and dephosphorylate the remaining dNTPs respectively (Figure 1). Specifically, 10 µl of PCR product were treated by adding 2 units of Exonuclease I (*Exo* I, New England Biolabs Inc., MA, USA), 15 units of Antarctic Phosphatase (New England Biolabs Inc., MA, USA), 1.5 µl of Antarctic Phosphatase buffer (10X) and 1.8 µl water. The enzymatic treatment was performed at 37°C for 90 min followed by incubation at 68°C for 45 min to deactivate the enzymes.

**Figure 3:**
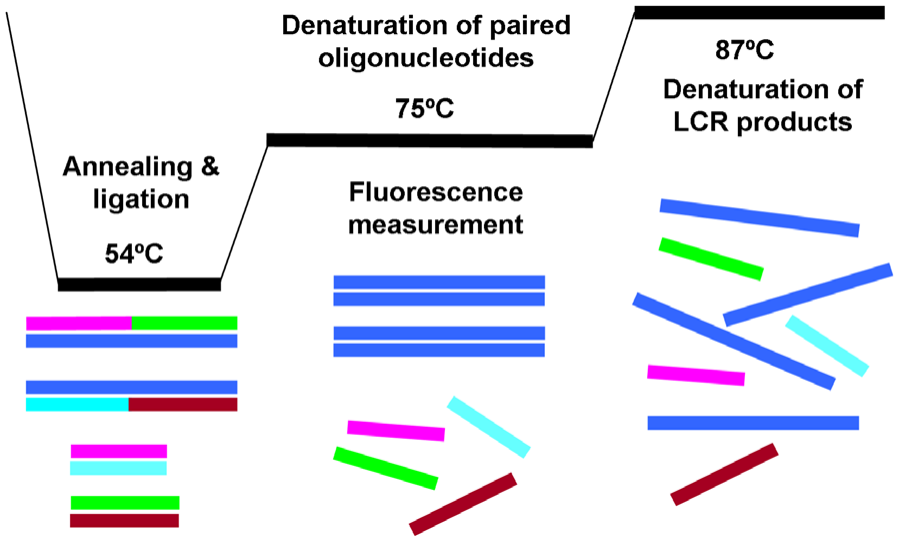
Schematic representation of the cycling steps for real-time LCR. LCR oligonucleotides are defined with colure purple for LCPR1, turquoise for LCPR2, green for LCPR3s and red for LCPR4s. PCR product used as LCR template is defined in deep blue.

Dilution (1/400) of the real-time PCR products in TE (10 mM Tris-HCl pH = 7.4) were used as templates for the ensuing real-time LCR. A schematic representation of the cycling steps for real-time LCR is shown in Figure 3. The process involved the use of two pairs of oligonucleotides, each consisting of a discriminating oligonucleotide and a 5′ phosphorylated oligonucleotide, which were complementary to one of the denatured target DNA strands (PCR product). During the annealing/ligation step the oligonucleotides annealed to the target DNA strands but also produced heterodimeres. Real-time monitoring was possible by utilizing a DNA-specific fluorescent dye and introducing a denaturation step for the paired oligonucleotides, at the highest possible temperature, where the LCR amplicons remained as heterodimers. A fluorescence measurement followed in order to quantify the amplicons produced in each cycle.

For the oligonucleotide design, initial melting simulations for each candidate LCR oligonucleotide and LCR-product with their respective complements were performed on the DINAMelt web server at http://www.bioinfo.rpi.edu/applications/hybrid/hybrid2.php [30]. Input parameters for Na^+^, Mg^++^ and oligonucleotide concentrations were 75 mM, 1.75 mM and 0.4 µM, respectively. Oligonucleotides LCPR1, LCPR2, LCPR3s, LCPR4s (Table 1), were designed for the basic real-time LCR protocol. Oligonucleotides LCPR1, LCPR4s were phosphorylated at their 5′-end. In order to avoid interference of oligonucleotide heterodimers with fluorescence measurements, care was taken to select oligonucleotide pairs which produce an LCR amplicon with high *T_m_* (i.e. 80°C), whereas their respective *T_m_* was at least 10°C less (Table 1). Another important issue addressed during oligonucleotide design was the avoidance of false ligation of the hybridized oligonucleotides on a mismatched wild-type DNA molecule during the paired oligonucleotides’ denaturation step. The possibility of these hybridization events to occur is reduced if the *T_m_* of the oligonucleotides is low. However, reduction of length for all 4 oligonucleotides would also reduce the *T_m_* of the LCR amplicon. Therefore in an effort to reduce false ligation during the paired oligonucleotides' denaturation step, only one out of the two oligonucleotides (either the discriminating or the 5′ phosphorylated one) able to hybridize on a mismatched wild-type DNA molecule was designed to have lower *T_m_,* i.e. LCPR3s and LCPR4s (Table 1).

For the basic protocol, two µl of PCR product were added to a 30 µl reaction mixture. The following reaction reagents were determined: 10 units of *Taq* DNA ligase (New England Biolabs Inc., MA, USA), 3 µl buffer (10X) from the *Taq* DNA polymerase buffer [200 mM Tris-HCl (pH = 8.4), 500 mM KCl] (Invitrogen, The Netherlands), 0.525 µl of respective *Taq* DNA ligase buffer (10X), 0.125 mM NAD^+^, 1.5 µl DNA-specific fluorescent dye EvaGreen™ (Biotium, Hayward, CA, USA) (20X), 0.4 µM of each LCPR1, LCPR2, LCPR3s, LCPR4s oligonucleotide and water up to 30 µl. During the setup all reagents and the 0.2 ml thin-wall tubes were kept in bench-top coolers. In addition, the *Taq*-ligase buffer was split into small aliquots in order to avoid multiple thawing that may affect the efficiency of the process.

Thermocycling was applied using the Mx3005P QPCR system (Stratagene Co., La Jolla, CA, USA). Specific conditions included three initial steps consisting of a PCR-amplicon denaturation step at 92°C (30 s), an annealing/ligation step at 54°C (6 s) and a second denaturation step at 92°C (30 s), followed by 39 LCR cycles, each one consisting of an annealing/ligation step at 54°C (6 s), a paired oligonucleotides' denaturation step at 75°C (30 s), where fluorescence measurement also was taken place, and an LCR product denaturation step at 87°C (20 s). At the end of amplification, a denaturation/renaturation cycle was included by heating at 95°C (5 min) followed by cooling at 74°C (3 min) to promote LCR-amplicon duplex formation, and at 37°C (1 min) for oligonucleotide duplex formation. A melting curve analysis followed. The EvaGreen™ was detected in the FAM channel using a gain setting of 8. The analysis of fluorescence data was conducted using the MxPro-Mx3005P software (Version 4.00; Stratagene, La Jolla, CA, USA). The baseline was adaptive and the threshold fluorescence levels used to derive threshold cycle (*Ct*) values were determined automatically. All individual samples were run in triplicates. Non-template controls containing all reagents minus the DNA template were included, to monitor the progress of template-independent ligation.

For the estimation of mutant DNA frequencies (%) standard curves were produced by plotting the threshold cycle values (*Ct* values) versus the logarithm of % frequency of mutant (V_136_) DNA in the standard plasmid DNA pools.

#### Alternative protocols

The basic protocol described above was termed protocol 1. Several variations to this protocol were also considered, based on changed parameters. These variations are summarized in Table 2. Specifically, protocols 2 to 6 were as protocol 1 but with different thermocycling conditions (duration of paired oligonucleotides' denaturation step or duration of annealing/ligation step or different paired oligonucleotides' denaturation step temperatures) (Table 2). Protocol 7 was a modification of protocol 1 in which the *Taq* DNA ligase and its respective buffer (10X) were replaced by 10 units of PFU ligase (Stratagene, La Jolla, CA, USA) and 0.525 µl PFU buffer (10X). Protocol 8 was a modification of protocol 1 in which LCPR3L and LCPR4L oligonucleotides were used instead of LCPR3s and LCPR4s. LCPR3s and LCPR4s were homologous to LCPR3L and LCPR4L but with different *Tm*. Protocols 9 and 10 were two modifications of protocol 1 in which nucleotide gaps were introduced between the adjacent oligonucleotides hybridized to the target DNA (PCR product). Protocol 9 was a “gap-T-LCR”, involving a thymine (T) gap between the adjacent hybridized oligonucleotides that corresponded to the C→T mutation of codon 136 of the *PRNP* gene. Protocol 10 was a “gap-A-LCR”, which involved a respective adenine (A) gap corresponding to the complementary strand of the DNA target. Both gap-LCR protocols (protocols 9 and 10) were run under the same reaction conditions as the protocol 1, except for the addition of 1.8 units of Platinum® *Taq* DNA polymerase (Invitrogen, The Netherlands) and 0.1 mM of the mutant-complementary insertion nucleotide; dTTP was used for gap-T-LCR and dATP for gap-A-LCR. In addition, in the case of gap-T-LCR (protocol 9), the LCPR2G oligonucleotide was used instead of LCPR2, whereas in the case of gap-A-LCR (protocol 10) the LCPR3G oligonucleotide replaced LCPR3s (Table 1).

**Table 2:** Different real-time LCR protocols tested.

Assay	DNA-Ligase	Duration of ligation	Duration of oligonucleotide denaturation	Temperature of oligonucleotide denaturation	Mean *Ct-*values of wild type samples (SD)	LOD^f^	LOQ^f^	Efficiency
Protocol-1^a^,^e^	Taq-DNA	6 s	30 s	75°C	25.02 (0.32)	0.085	0.35	80%
Protocol- 2^a^	Taq-DNA	6 s	35 s	75°C	23.71 (0.40)	0.16	0.85	82%
Protocol-3^a^	Taq-DNA	6 s	40 s	75°C	23.1 (0.38)	0.23	1.13	81%
Protocol-4^a^	Taq-DNA	12 s	30 s	75°C	23.24 (0.41)	0.15	0.88	83%
Protocol-5^a^	Taq-DNA	24 s	30 s	75°C	20.49 (0.34)	0.21	0.99	82%
Protocol-6^a^	Taq-DNA	6 s	30 s	74°C	25.56 (0.55)	0.097	1.14	70%
Protocol- 7^a^	PFU-DNA	6 s	30 s	75°C	30.10 (0.41)	0.098	0.39	70%
Protocol-8^b^	Taq-DNA	6 s	30 s	75°C	26.40 (0.32)	0.34	1.07	83%
Protocol- 9^c^	Taq-DNA	6 s	30 s	75°C	52.12 (0.56)	0.14	0.40	31%
Protocol- 10^d^	Taq-DNA	6 s	30 s	75°C	37.39 (0.30)	0.11	0.34	51%

Different thermocycling conditions, DNA ligases, length of oligonucleotides, gap modifications were examined. All trials were performed with the Mx3005P QPCR platform. Gap-A and gap-T LCR protocols used Platinum® *Taq* DNA polymerase.

a Oligonucleotides used LCPR1, LCPR2, LCPR3s, LCPR4s.

b Oligonucleotides used LCPR1, LCPR2, LCPR3L, LCPR4L.

c Oligonucleotides used LCPR1, LCPR2G, LCPR3s, LCPR4s.

d Oligonucleotides used LCPR1, LCPR2, LCPR3G, LCPR4s.

e Optimal real-time LCR protocol.

f The LOD and LOQ values are in % frequencies

### Statistical Analysis

#### Detection and quantification limits

All protocols described above were evaluated based on limits of detection (LOD) and limits of quantification (LOQ) of V_136_, as defined in [31]. Specifically, the LOD was defined as the lowest ratio of mutant to wild-type DNA that can be determined to be different from the wild-type DNA, whereas the LOQ can be defined as the lowest ratio of mutant to wild-type DNA above which quantitative results may be obtained [31].

In the present study, LOD and LOQ were determined using the *Ct* values obtained from the wild-type templates of 25 individual samples. These *Ct*-values were first tested for normality using the Kolmogorov-Smirnov and Shapiro-Wilk tests. Both tests showed that *Ct-*values followed a normal distribution. Subsequently, LOD and LOQ were calculated according to the following formulae:
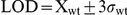


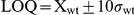
where X_wt_ is the mean *Ct* value and σ_wt_ is the standard deviation of the wild-type DNA *Ct* measurements. In practical terms, LOD was set at the concentration that gives a signal equal to three times the standard deviation of the wild-type sample measurements [32]. The three-standard deviation criterion represents a 99.73% probability that the value is above the background level. Similarly, LOQ was set at ten times the standard deviation of the wild-type sample measurements, representing a corresponding probability higher than 99.99% [32]. Finally, LOD and LOQ were expressed as mutant-type frequencies (%) by fitting a standard curve generated by the standard plasmid DNA pools.

Further protocol comparisons were made between average *Ct-values* from the analysis of samples with the lowest known mutant (0.39% V_136_) frequency, used for the determination of the standard curves, with samples with 100% wild-type (A_136_) frequency. A t-test was used in this regard. All protocols shown in Table 2 were tested.

#### Linearity and efficiency

Linearity and efficiency of the protocols were assessed using a standard curve constructed by the amplification of plasmid standard pools with a total number of 5·10^9^ copies/ml including different mutant DNA frequencies, ranging from 100% to 0.39%. Efficiency (E) was calculated using the equation: E = 10 ^(−1/slope)^ -1; slope was estimated directly by the MxPro-Mx3005P software (Version 4.00; Stratagene, La Jolla, CA, USA).

#### Determination of reproducibility

To assess the reproducibility of the basic real-time LCR protocol, the intra-assay and inter-assay coefficients of variation (CV) were evaluated, based on the measured mutant V_136_ frequencies (%) in the DNA pools. For intra-assay CV calculation, standard plasmid pools were applied in eight replicates at each concentration level of the mutant V_136_, in one trial run. For inter-assay CV calculation, the plasmid standard pools were applied three-fold in three different trial runs performed on different days using freshly prepared reagents.

#### Accuracy of quantification using genomic DNA pools and natural samples of individual animals

The accuracy of the basic real-time LCR protocol for mutant DNA quantification was assessed by testing the genomic DNA pools prepared from extracted samples containing the V_136_ polymorphism at different pre-determined frequencies (50%, 25%, 6.25%, 1.56%, and 0.39%). For each frequency, four different DNA pools were tested. Furthermore, each of these pools was tested in four replicates and the V_136_ frequency was calculated by plotting the mean *Ct* value in a standard curve created by the plasmid standard pools (100%, 25%, 6.25%, 1.56%, and 0.39%) that were included in the same trial. A linear regression analysis of the estimated mutant (V_136_) log_10_ frequencies on the actual (known) log_10_ frequencies was performed to test the quantification accuracy.

In addition, all 60 individual animal samples that had been genotyped with RFLP analysis were re-tested with the basic real-time LCR protocol in order to detect and quantify V_136_ mutant DNA.

## Results and Discussion

### Real-time PCR amplification and treatment with Exonuclease I and Antarctic Phosphatase

The first step introduced was a real-time PCR, which amplified a genomic region containing the SNP in order to develop the template needed for the ensuing LCR protocols. DNA quantification became possible using the intercalating DNA dye, EvaGreen [28], [29]. Previously, in a real-time PCR platform the intensity of fluorescence was found to be proportional to the total amount of the DNA in the tube [28]. In the present study, real time PCR amplification curve plateaus indicated that equimolar PCR products were amplified, as approximately 99.9% of the samples gave fluorescence plateau intensities with low variability (CV = 11%), irrespectively of their original DNA concentrations; the latter were substantially different, as evidenced by their respective *Ct* values that ranged from 26.1 to 30.9 cycles, suggesting a difference in DNA quantity of up to 27.8-folds (efficiency 100%) (Figure 2). A very low number of samples, approximately 0.1%, showed plateaus outside the ±3 SD range from the mean and were not included in the ensuing LCR trials. Thus, with the incorporation of this real-time PCR step, the vast majority of samples could be subsequently tested reliably in the real-time LCR, irrespectively of their original DNA concentration.

Furthermore, in order to assess the effect of different concentrations of extracted DNA on the final real-time LCR results, 3 serial 10-fold dilutions of a heterozygous A_136_/V_136_ genomic DNA extract were tested; DNA extract dilutions had been prepared using TE (10 mM Tris-HCl, pH = 7.4). Real-time amplification results showed different *Ct* values, as expected due to different dilutions, but similar fluorescence plateaus (data not shown). In all cases, the basic real-time LCR protocol determined the correct (50%) percentage of V_136_, showing that the method is not dependent on the initial DNA concentration of the extract. Hence, the method may be applicable to cases where minute amounts of DNA are available (e.g. forensics, cancer biopsies with limited number of cells etc).

In addition, the necessity of PCR-product treatment with Antarctic Phosphatase and Exonuclease I was demonstrated by running LCR trials using real-time PCR products that had not been previously treated with these enzymes (data not shown). The samples that had not been subjected to this treatment, showed very high *Ct* variability and low mean-*Ct* value compared to the treated samples. Lower *Ct* values obtained from PCR products that had not been treated with Antarctic phosphatase and Exonuclease I could be attributed either to polymerization events during the LCR due to the presence of residual *Taq* polymerase and dNTPs, or possible presence of ssDNA amplicons that could provide a template for false ligation events during the LCR setup. Both events were avoided by the simultaneous treatment with Antarctic phosphatase and Exonuclease I, which permitted the dephosphorylation of residual dNTPs and degradation of PCR primers along with potential ssDNA amplicons. Therefore the need for incorporating a laborious purification step of the PCR product was alleviated.

### Evaluation of the method for SNP detection and quantification

#### Impact of different parameters on detection and quantification limits

**Figure 4:**
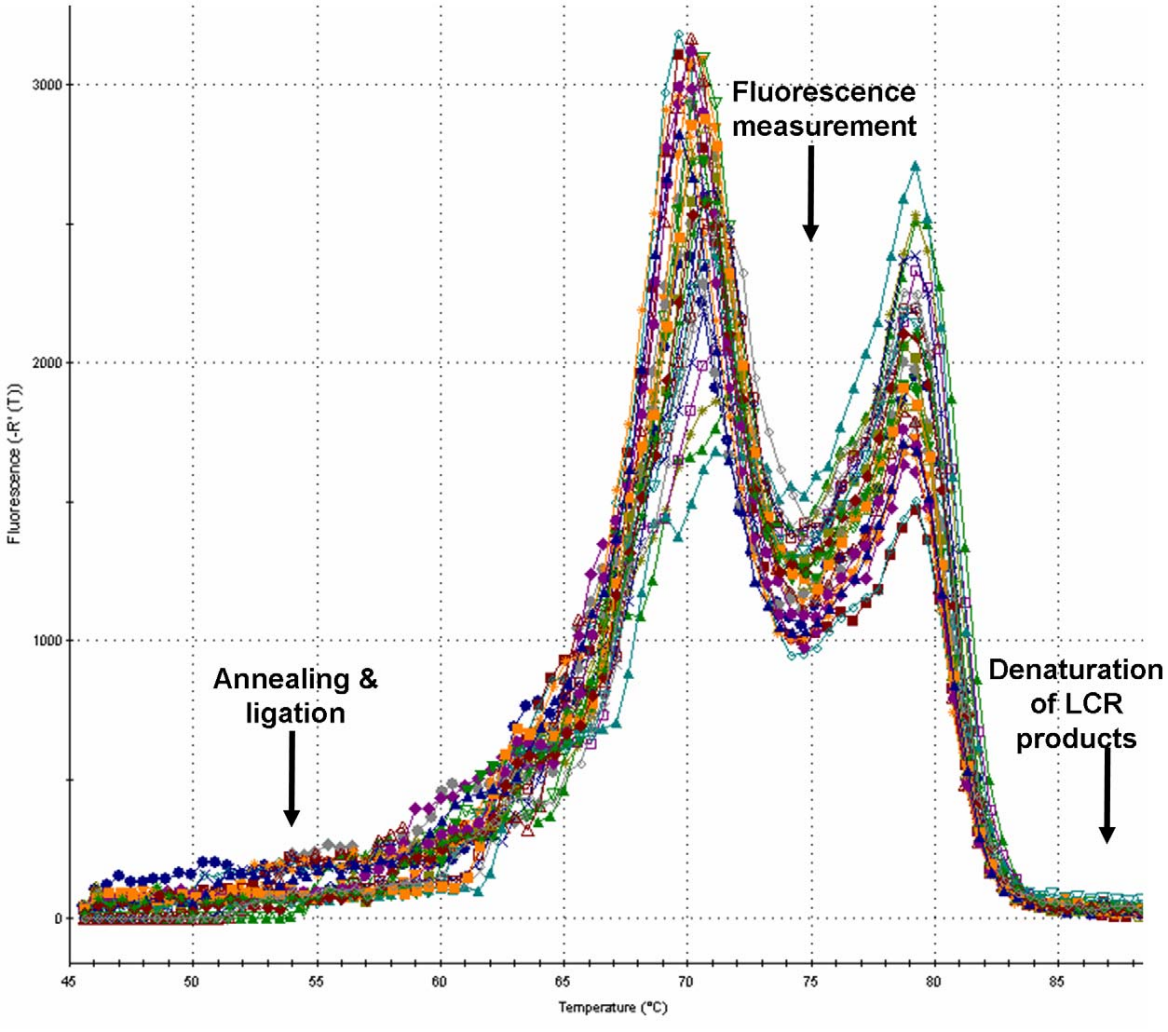
Melting curve profile of the LCR products. The first peak represents the melting of oligonucleotide dimers and the second the melting of LCR products. Selected temperatures for LCR steps are indicated by arrows.

Results pertaining to the impact of the different parameters, as illustrated by the ten protocols, are shown at Table 2. It became obvious that the extent of SNP discrimination depended mainly on the thermocycling conditions. Trials with different thermocycling parameters (Protocols 1 to 6) showed that the duration of annealing/ligation and paired oligonucleotides' denaturation steps, along with the temperature of paired oligonucleotides' denaturation step, were determining factors for the specificity of ligation. These parameters changed the *Ct* values of the wild-type samples and, therefore, LOD and LOQ were affected. Higher time duration of annealing/ligation and paired oligonucleotides' denaturation steps, increased (worsened) both LOD and LOQ (Table 2). These results could be attributed to non-specific ligation events during these steps. Such events can occur due to false ligation of oligonucleotides hybridized on mismatched wild-type DNA molecules [19] as well as sticky-end ligation of the paired oligonucleotides [18]. Both non-specific ligation events pose limitations on the LOD and LOQ of the real-time LCR because they lead to non-specific background signals. Therefore, short time period for both steps is crucial. Minimum duration of annealing/ligation (6 s) and paired oligonucleotides' denaturation (30 s) steps (protocol 1) proved to be the optimum for obtaining low LOD and LOQ values. Additional control trials showed that, when the paired oligonucleotides' denaturation step temperature was reduced from 75°C (protocol 1) to 74°C (protocol 6), the LOD and LOQ values were considerably increased (Table 2). At lower oligonucleotides' denaturation step temperatures than the proposed one (75°C), the ligase has more time to exhibit non-specific activity due to the presence of oligonucleotides hybridized on mismatched wild-type DNA molecules. The initial selection of temperatures for real-time LCR cycling steps was based on melting simulations for each candidate LCR oligonucleotide and LCR product, with their respective complements. The most important criteria for temperature selection were maximum concentrations of LCR amplicon heterodimers and minimum concentrations for oligonucleotide heterodimers during fluorescence measurement (Figure 4). Nevertheless, as results of the present study illustrated, delicate adjustments of temperature and duration of this step are important for optimum sensitivity of SNP discrimination in DNA pools.

In an effort to reduce false ligation during the paired oligonucleotides' denaturation step, one out of the two oligonucleotides able to hybridize on a mismatched wild-type DNA molecule (i.e. LCPR3s and LCPR4s) was designed to have lower *T_m_.* It was expected that these shorter oligonucleotides, would not be able to efficiently hybridize on either strand of the target-DNA during the paired oligonucleotides' denaturation step and therefore contribute to the decrease of LOD and LOQ. This hypothesis was supported after substitution of LCPR3s and LCPR4s (protocol 1) with the homologous longer oligonucleotides LCPR3L and LCPR4L (protocol 8) (Table 1) which resulted in an almost three-fold increase of LOD and LOQ (Table 2).

Moreover, substitution of *Taq* DNA ligase (protocol 1) by PFU thermostable ligase (protocol 7) did not lead to any considerable improvement regarding LOD and LOQ values (Table 2). This result was contrary to others previously reported, which suggested that PFU DNA ligase had higher fidelity than *Taq* DNA ligase [33].

In the present study calculated LOD and LOQ values for both protocols 9 and 10 (gap-T and gap-A) were not better than those of the protocol 1 (Table 2). Only the LOQ calculated for protocol 10 was slightly lower (0.34 versus 0.35). The theoretical advantage of gap-LCR is the avoidance of sticky-end ligation [18]. This is explained by an initial extension of the discriminating oligonucleotide by a DNA polymerase which precedes the ligation process. According to the present results, reduction of sticky-end ligation was also possible in the basic protocol (protocol 1) due to performing the setup at low temperatures by keeping reagents and thin wall tubes in bench-top coolers during the real-time LCR setup. This observation was supported by some preliminary real-time LCR trials (not shown) with a setup at room temperature which led to an increase of sticky-end ligation, as indicated by decreased *Ct* values of the non-template controls.

Further gap-LCR trials were performed with proofreading polymerases [PFX Platinum (Invitrogen, The Netherlands), Phusion® (New England Biolabs Inc., MA, USA) and Vent® (New England Biolabs Inc., MA, USA)] in an effort to improve the SNP discrimination capability of the LCR (results not shown). These enzymes exhibit a 3′→5′ exonuclease proofreading activity. It was expected that proofreading activity at the site of point mutation would exclude the incorporation of a mismatched nucleotide during the gap filling and, thus, improve the specificity of gap-LCR applications. In this case, phosphorothioate (PTO) and locked nucleic acid (LNA) modifications of LCR oligonucleotides were also introduced to avoid degradation of their 3′ termini [34], [35]. However, PTO modifications led to increased *Ct*-variability and LOD, possibly due to interference with the catalytic domain of the ligase in a way that impairing the fidelity of the process. Furthermore, LNA modifications resulted in partial oligonucleotide degradation and sample amplification failure. These results demonstrate that proofreading polymerases are not suitable for real-time gap-LCR protocols.

Comparisons made between average *Ct-values* from the analysis of samples with the lowest known mutant (0.39% V_136_) frequencies and 100% wild-type (A_136_) samples, showed that differences were statistically significant (P<0.05) for all protocols except from protocol 8 (which used different oligonucleotides). When significant, results meant that V_136_ detection was possible at this frequency. The lowest P-value was observed for protocol 1 (P = 0.001), followed by protocols 9 and 10 (P = 0.003 and 0.002). This result suggested that, in 999 in 1,000 cases, protocol 1 would effectively detect V_136_, when the frequency of the latter was 0.39%. In practical terms, detection of this deleterious mutation would be possible in a flock of sheep, using their pooled DNA, even when only 1 in 250 animals was carrier of the mutant DNA.

#### Linearity and Efficiency

Real-time LCR protocols 1 to 8 showed similar efficiency at the satisfactory level of 80% (Table 2). The absolute magnitude of this parameter is difficult to assess due to lack of literature reports on LCR efficiency. The possible explanation for the observed, lower than 100% LCR amplification efficiency is the formation of oligonucleotide pairs during the annealing/ligation step, which reduces their availability for annealing to the target-DNA. For both gap-LCR protocols (protocols 9 and 10), the amplification efficiency was much lower compared to the others (Table 2). The complexity of gap-LCR necessitating the simultaneous activity of both enzymes (ligase and polymerase) is the probable cause of this reduction in efficiency.

**Figure 5:**
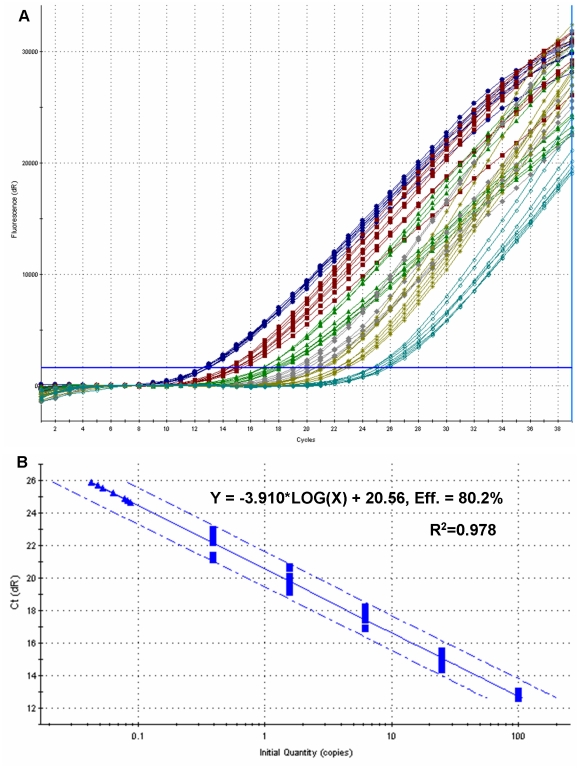
Amplification plots of mutant DNA (V_136_) detection in plasmid standard pools. (A) FAM fluorescent signals and (B) corresponding standard curve generated from plasmid standard pools with pre-defined mutant DNA concentrations. From left to right, curves represent 100%, (blue lines with circles), 25% (red lines with squares), 6.25% (green lines with triangles), 1.56% (grey lines with diamonds) and 0.39% (yellow lines with stars), V_136_ frequencies (the 95% confidence limits for the standard curve are shown as hashed lines). Wild-type (A_136_) samples are also included in the reaction and are placed in the far right (A) and are denoted by triangles in (B). Reactions were performed with 8 replicates. The threshold cycle (*Ct*) values (B) are plotted against the logarithm of the mutant DNA frequency (%).

In all protocols, there was a linear relationship between *Ct* values and V_136_ frequencies, manifested by a very high (>0.97) R^2^ value of the regression of the latter on the former (Figure 5). In all cases, regression slopes were very close to unity and intercepts were practically zero, attesting to the high accuracy of quantification.

Based on LOD, LOQ, linearity and efficiency was concluded that all variations introduced did not further improve the basic protocol. Therefore, the latter (protocol 1) is our protocol of choice. Only protocol 10 improved slightly LOQ, but since it required additional consumables (*Taq* DNA polymerase), it is not recommended.

#### Determination of reproducibility

The experimental intra- and inter-assay variability obtained using plasmid standard pools with different V_136_ frequencies is summarized in Table 3. Good reproducibility is an essential requirement of quantitative assays. The low intra- and inter-assay variability of the basic real-time LCR protocol obtained in the present study is comparable with other quantitative DNA amplification methods based on real-time PCR [36].

**Table 3:** Experimental intra- and inter-assay variability of the basic real-time LCR protocol.

Variation	Frequency of V_136_	CV (%)
**Intra-assay**	100%	11.29
**Intra-assay**	25%	21.45
**Intra-assay**	6.25%	26.17
**Intra-assay**	1.56%	26.54
**Intra-assay**	0.39%	18.03
**Inter-assay**	100%	15.32
**Inter-assay**	25%	15.97
**Inter-assay**	6.25%	17.00
**Inter-assay**	1.56%	12.80
**Inter-assay**	0.39%	9.47

The coefficient of variation (CV) was calculated from the measured mutant V_136_ frequencies in the plasmid standards.

#### Accuracy of quantification using genomic DNA pools and natural samples of individual animals

**Figure 6:**
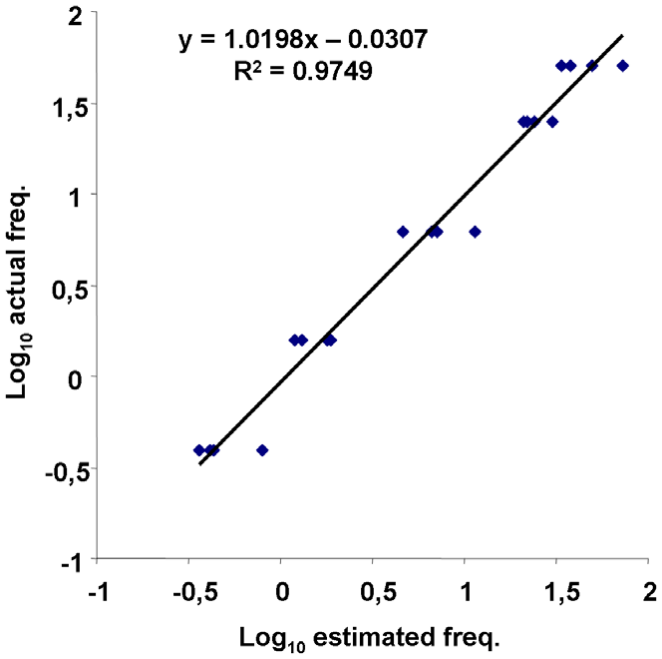
Linear regression of the estimated mutant (V_136_) log_10_ frequencies on the actual log_10_ frequencies. Four series of genomic DNA pools containing V_136_ polymorphism at different frequencies (50%, 25%, 6.25%, 1.56%, and 0.39%) were tested by the basic real-time LCR protocol. Each sample was analysed in four replicates.

The ability of the basic real-time LCR protocol to accurately quantify mutant DNA was assessed using genomic DNA pools that were artificially prepared to contain different mutant DNA frequencies (50%, 25%, 6.25%, 1.56%, and 0.39%). Estimates of the V_136_ frequencies were calculated by plotting the mean *Ct* values in a standard curve created by plasmid standard pools. Linear regression analysis of the estimated mutant (V_136_) log_10_ frequencies on the actual (known) log_10_ frequencies showed a high linear relationship between the two sets of values (R^2^ = 0.9749, Figure 6). Slope and intercept of the regression were not significantly different from 1 and 0, respectively (P>0.05). These results attest to the predictive capacity of the basic real-time LCR protocol developed in the present study. Furthermore, these results are comparable to those obtained with plasmid standards, described previously, suggesting that the method is quantitative at both types of pools. This is the first time, to our knowledge, that a quantitative LCR protocol was applied to the quantification of mutant DNA at the full range of LCR sensitivity.

All 60 genotyped individual animal samples were re-tested with the basic real-time LCR protocol in order to detect and quantify of V_136_ mutant DNA. With the basic real-time LCR protocol it was possible to determine the individual animal genotypes in all sixty milk samples. One animal was homozygous V_136_, five were heterozygous V_136_ and 44 were homozygous A_136_. The mean (standard deviations in parenthesis) *Ct* value obtained for the homozygous V_136_ sample was 15.06, for the heterozygous V_136_ samples was 16.75 (0.35) and for the homozygous A_136_ samples was 28.34 (0.64). The percentage of V_136_ calculated by the standard curve created using plasmid standard pools was 111% for homozygous V_136_ and 47.03% for the heterozygous V_136_ samples. Moreover, these genotyping results were consistent with RFLP genotyping of the same animals, which in turn was verified by sequencing 21 of them (data not shown). Therefore, the basic real-time LCR protocol developed in the present study proved suitable for individual genotyping, too.

#### Application in an alternative real-time platform

In addition to the Mx3005P QPCR system (Stratagene Co., La Jolla, CA, USA), the basic real-time LCR protocol was also evaluated in a Mini Opticon™ System (Biorad, Milan, Italy). LOD and LOQ values, efficiency and linearity of the assay were similar in the two systems. Nevertheless, the optimum time of oligonucleotides' denaturation step for the second thermocycler was 15 s, instead of 30 s in Mx3005P QPCR. As discussed above, this may be attributed to different temperature stabilization times required by the two systems for accurate fluorescent measurements. Furthermore, the optimum dilution of real-time PCR product used as target DNA was 1/100 (compared to 1/400 in the first system). This may be due to different software requirements between the two systems in establishing the baseline.

The utility of this result lies on the fact that the basic real-time LCR protocol developed in the present study is applicable to different platforms, although minor adjustments might be needed. However, such adjustments can be made by the user. This is an advantage of the present method over the semi-quantitative protocol proposed previously [19], which required intervention by the system manufacturer to modify the optics configuration.

### Conclusion

A real-time LCR protocol was developed for accurate SNP detection and quantification in DNA pools. This method offers several important advantages that include high sensitivity, excellent specificity and reproducibility. In addition, a large number of samples can be processed. The latter is important since the main aim of this study was the development of a practical method for large-scale assessment of the prevalence of human or animal SNP in DNA pools. A model application of the method is the quantification of *PRNP* polymorphisms, such as the undesirable V_136_ in bulk milk, in order to quantify the scrapie risk in ovine milk and its dairy products. This process may enable labelling and marketing of potentially ‘scrapie free’ dairy products. The value of a system able to sensitively detect prion protein mutations on DNA pools is of particular interest, since increasingly strict regulations on food safety and public hygiene require new, practical and animal friendly methods for large scale implementation.

The real-time LCR protocol developed in the present study has high potential for future use in automated, high throughput formats, which could allow application in a wide range of interests including detection and quantification of oncogenes or tumour suppressor genes, infectious diseases, pathogenic bacteria, fungal species, viral mutants, drug resistance resulting from point mutations, genetically modified organisms in food and QTL.
